# The Global Diversity of Parasitic Isopods Associated with Crustacean Hosts (Isopoda: Bopyroidea and Cryptoniscoidea)

**DOI:** 10.1371/journal.pone.0035350

**Published:** 2012-04-25

**Authors:** Jason D. Williams, Christopher B. Boyko

**Affiliations:** 1 Department of Biology, Hofstra University, Hempstead, New York, United States of America; 2 Department of Biology, Dowling College, Oakdale, New York, United States of America; 3 Division of Invertebrate Zoology, American Museum of Natural History, New York, New York, United States of America; Utah State University, United States of America

## Abstract

Parasitic isopods of Bopyroidea and Cryptoniscoidea (commonly referred to as epicarideans) are unique in using crustaceans as both intermediate and definitive hosts. In total, 795 epicarideans are known, representing ∼7.7% of described isopods. The rate of description of parasitic species has not matched that of free-living isopods and this disparity will likely continue due to the more cryptic nature of these parasites. Distribution patterns of epicarideans are influenced by a combination of their definitive (both benthic and pelagic species) and intermediate (pelagic copepod) host distributions, although host specificity is poorly known for most species. Among epicarideans, nearly all species in Bopyroidea are ectoparasitic on decapod hosts. Bopyrids are the most diverse taxon (605 species), with their highest diversity in the North West Pacific (139 species), East Asian Sea (120 species), and Central Indian Ocean (44 species). The diversity patterns of Cryptoniscoidea (99 species, endoparasites of a diverse assemblage of crustacean hosts) are distinct from bopyrids, with the greatest diversity of cryptoniscoids in the North East Atlantic (18 species) followed by the Antarctic, Mediterranean, and Arctic regions (13, 12, and 8 species, respectively). Dajidae (54 species, ectoparasites of shrimp, mysids, and euphausids) exhibits highest diversity in the Antarctic (7 species) with 14 species in the Arctic and North East Atlantic regions combined. Entoniscidae (37 species, endoparasites within anomuran, brachyuran and shrimp hosts) show highest diversity in the North West Pacific (10 species) and North East Atlantic (8 species). Most epicarideans are known from relatively shallow waters, although some bopyrids are known from depths below 4000 m. Lack of parasitic groups in certain geographic areas is likely a sampling artifact and we predict that the Central Indian Ocean and East Asian Sea (in particular, the Indo-Malay-Philippines Archipelago) hold a wealth of undescribed species, reflecting our knowledge of host diversity patterns.

## Introduction

**Figure 1:**
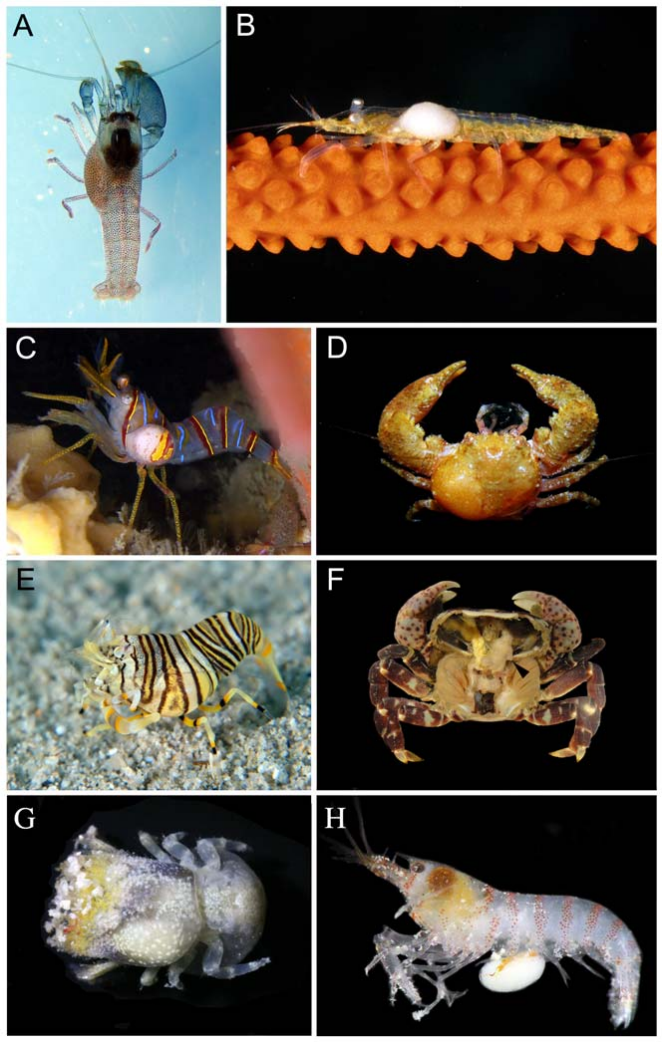
Representative crustacean hosts parasitized by epicaridean isopods. A) *Synalpheus fritzmuelleri* Coutière, 1909 (Caridea: Alphaeidae) from Caribbean Panama, with unidentified bopyrid, possibly *Bopyrella harmopleon* Bowman, 1956 (known from this host in Venezuela); B) *Miopontonia yongei* Bruce, 1985 (Caridea: Palaemonidae) from Bali, Indonesia (new host locality record, previously known from Australia and southern Japan), with unidentified bopyrid (host previously not recorded with any bopyrid); C) *Lebbeus grandimanus* (Bražnikov, 1907) (Caridea: Hippolytidae) from North East Pacific, with unidentified bopyrid, likely *Bopyroides hippolytes* Krøyer, 1838 (known from this host in the North East Pacific); D) *Aliaporcellana* cf. *suluensis* (Anomura: Porcellanidae) from Fiji possibly with *Allorbimorphus haigae* Bourdon, 1976 (known from this host in Indonesia but this Fijian would represent a more than 5600 km range extension for this bopyrid); E) *Gnathophyllum americanum* Guérin-Méneville, 1855 (Caridea: Gnathophyllidae) from Japan with unidentified bopyrid, possibly *Schizobopyrina bombyliaster* Williams & Boyko, 2004 (known from this host in Tonga); F) *Hemigrapsus nudus* (Dana, 1851) (Brachyura: Varunidae) from Coos Bay, Oregon with carapace removed to show the entoniscid *Portunion conformis* Muscatine, 1956 (arrowhead); G) *Lithoscaptus helleri* (Fize & Serene, 1957) (Brachyura: Cryptochiridae) from Indonesia with unidentified bopyrid in left branchial chamber (host previously not recorded with any bopyrid); H) *Alpheus* sp. aff. *paracrinitus* Miers, 1881 (Caridea: Alpheidae) from Fiji with *Faba* sp. (Cryptoniscidae) attached to ventral surface (host not previously recorded with any epicaridean). Photographs used by permission of Arthur Anker (A, D), Rokus Groeneveld (B), Bob Bailey (C), Yoshihisa Fujita (E), Jason Williams (F), Sancia van der Meij (G) and Leslie Harris (H).

Within crustaceans, a wide range of groups including amphipods, barnacles, copepods, and isopods have formed parasitic relationships with invertebrate and vertebrate hosts. Three taxa within Isopoda (Bopyroidea, Cryptoniscoidea and Cymothooidea, including Gnathiidae) are composed of parasites that attach either permanently or during larval stages to their hosts (some cymothooids such as Aegidae are temporary ectoparasites or micropredators of fish). Cymothooids (∼1250 species) are ectoparasites of fish, with most cymothooids feeding on the blood and tissue of hosts as larvae and adults whereas gnathiids only parasitize fish during larval stages [1], [2]. In contrast, bopyroids and cryptoniscoids (795 species) are unique in that they use crustaceans as both intermediate and definitive hosts (Figure 1A–H). The isopods that parasitize crustacean hosts comprise approximately 7.7% of all isopods (estimated at 10,300 species; [3]) and are the focus of this review.

Paleontological evidence shows bopyroids and cryptoniscoids (commonly referred to as epicarideans; see [4]) have a shared evolutionary history with hosts dating back to at least the Jurassic [5]. Based on molecular and morphological evidence, epicarideans appear to be derived from a cymothoid-like (fish parasite) ancestor and this evolutionary host switch allowed for the radiation of species seen today [6]. At present, epicarideans are represented by Bopyroidea with three families (Bopyridae, Dajidae, Entoniscidae) and Cryptoniscoidea that contains seven families (Asconiscidae, Cabiropidae, Crinoniscidae, Cryptoniscidae, Cyproniscidae, Hemioniscidae, and Podasconidae) [7], [8]. The phylogenetic relationships between and within the higher taxa of epicarideans (particularly cryptoniscoids) are poorly known [6]. Molecular studies indicate that the group is in need of extensive revision, with Bopyroidea potentially being non-monophyletic (Boyko et al., in prep.). Because most bopyrids and dajids are ectoparasitic macroparasites, they are relatively easy to collect and observe on hosts. Therefore, they hold promise for analysis of host/parasite relationships and could be useful in co-evolutionary studies [4].

**Figure 2:**
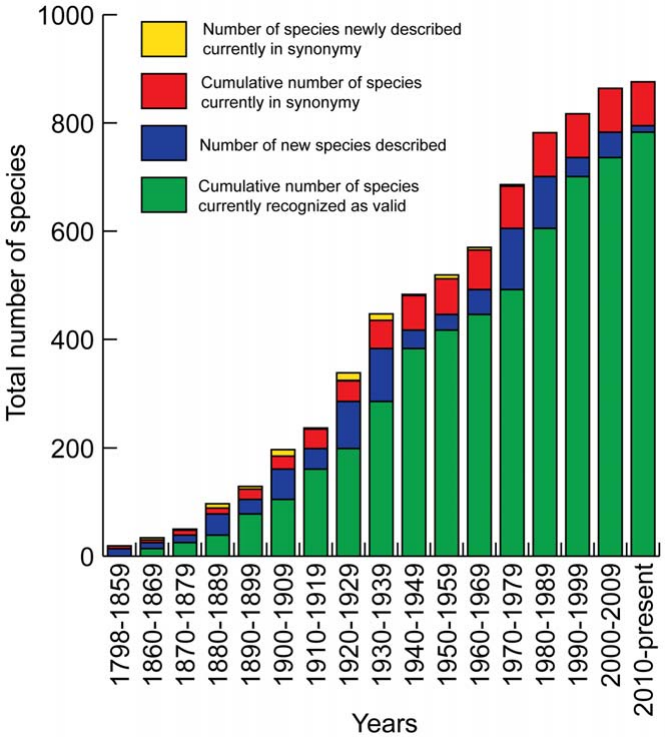
Number of epicaridean species described over time from 1798 (description of the first named epicaridean by Fabricius) to the present with the number of species newly described during each date range in blue (those currently regarded as valid) and yellow (those now considered as synonyms). Cumulative numbers of species described over time are in green (those currently regarded as valid) and red (those now considered as synonyms).

Epicarideans have a long history of taxonomic study dating back to 1724 and the first mention in the literature as a supposed larva of a flatfish parasitizing a shrimp [9] and the subsequent naming of the first bopyrid species in 1798 [10]. Research on this group has progressed in an irregular fashion, with short bouts of vibrant activity interspersed with longer, less active periods (Figure 2). No one has completed a comprehensive review of the global diversity of parasitic isopods associated with crustacean hosts (although regional reviews exist for some groups; e.g., [11], [12], [13]). The last review of the most speciose family (Bopyridae) was completed over 25 years ago [5], and serves an excellent benchmark to show how studies on the taxonomy and biogeography of the group have progressed.

## Methods

Data were gathered from the literature since 1864 (inception of the *Zoological Record*), online sources [14], and our personal records. Most described species are included in the analyses (but see below), including those species later entered into synonymy, as well as the still recognized enigmatic and monotypic families Colypuridae Richardson, 1905 and Rhabdochiridae Richardson, 1905 (Bopyroidea), which are based on a single male and a single larval form, respectively. We excluded all *nomina nuda*, all “species” of *Microniscus* (a genus erected for larval forms from copepod hosts), any replacement names for homonyms, and *Proteolepas bivincta* Darwin, 1854 (which may be an epicaridean but is presently considered a *nomen dubium*). Rate of species descriptions over time is reported by decade, excepting the years 1798–1859, where 1 to 7 species per decade were described and these data are therefore combined. Total number of described species and genera were reported for the biogeographic regions (“Marine Regions”) of [15], which are similar to the regions defined by the National and Regional Implementation Committees (NRIC) [16]. We analyzed the data for these regions, rather then other biogeographic conceptions (e.g.. [17]) having more fine-grained divisions, due to the lack of sufficient sampling in many of the smaller subdvisions that would give misleading “patterns” of distribution for bopyrids.

## Results and Discussion

### Rate of species descriptions

The rate of species description of epicarideans has been inconsistent, with two clear peaks of activity in the 1920s and 1930s (largely thanks to the efforts of Nierstrasz & Brender à Brandis and Shiino) and again in the 1970s and 1980s (primarily the work of Bourdon and Markham) (Figure 2). The earliest years of description (1798–1869) resulted in relatively few species (34) with approximately 26% of them later being relegated to synonymy. In comparison, the past 31 years (1980–2011) have not seen any new species described in this time interval placed into synonymy. The per-decade rate of species descriptions is ∼41 (excluding the incomplete decades of 1790s and 2010s). Overall, 9.3% of all species described have been synonymized.

### Fossil record

Parasitization of decapods by bopyrids is seen in the fossil record and extends back to at least the Jurassic [5], including several records in squat lobsters (Galatheoidea, see [18], [19]), a taxon thought a likely candidate for being the first target of epicaridean parasitism in decapods. Identification beyond recognition of a bopyrid presence in fossils is impossible, as only the characteristic swelling of host branchial chambers exists and no species of fossil bopyrids have ever been described. Interestingly, some decapod families are known with these parasites on extant species but not as fossils, whereas other families with numerous records of fossil parasites, such as Raninidae (Brachyura), apparently have no bopyrids on extant species [20]. No fossil record exists for any epicarideans outside of the Bopyridae, which is to be expected given the general lack of external host modifications from non-bopyrid taxa, although some entoniscids and cryptoniscoids cause slight deformations in hosts [21], [22], [23], [24].

### Morphology and definitive hosts

The morphology of epicarideans can be quite modified from free-living isopods, and females of some taxa are scarcely recognizable as being isopods. Although loss of structures in derived taxa may occur (e.g., number of pereopods, pleopods), it cannot be stated that all aspects of epicaridean morphology display such reduction. In fact, many species have highly specialized features developed for aid in attachment to hosts, such as the dactyl sockets of species in *Asymmetrione* (Pseudioninae) and attachment suckers on the oostegites in some species of Hemiarthrinae. All epicaridean species have sexual dimorphism, with larger females having more modified features than the dwarf males.

**Figure 3:**
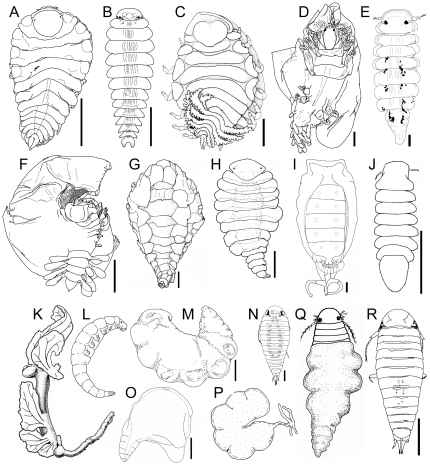
Representative bopyroid and cryptoniscoid isopods, a selection of body forms. A) *Pseudione quasimodo* Boyko & Williams, 2004 (Bopyridae: Pseudioninae), female dorsal view; B) *Pseudione quasimodo*, male dorsal view; C) *Dactylokepon semipennatus* Bourdon, 1983 (Bopyridae: Ioninae), female dorsal view; D) *Minimathelges nanus* Boyko & Williams, 2003 (Bopyridae: Athelginae), female dorsal view; E) *Minimathelges nanus*, male dorsal view; F) *Hemiarthrus surculus* Boyko & Williams, 2004 (Bopyridae: Hemiarthrinae), female dorsal view; G) *Entophilus mirabiledictu* Markham & Dworschak, 2005 (Bopyridae: Entophilinae), female dorsal view; H) *Entophilus mirabiledictu*, male dorsal view; I, *Heterophryxus appendiculatus* G. O. Sars, 1885 (Dajidae), female dorsal view, with male attached; J) *Heterophryxus appendiculatus*, male dorsal view; K) *Paguritherium alatum* Reinhard, 1945 (Entoniscidae), female lateral view, with enclosing sheath removed. L) *Paguritherium alatum*, male lateral view; M) *Cabirops bombyliophila* Williams & Boyko, 2004 (Cabiropidae), female lateral view; N) *Cabirops bombyliophila*, male dorsal view; O) *Crinoniscus politosummus* Hosie, 2008 (Crinoniscidae), female lateral view; P) *Danalia curvata* (Fraisse, 1878) (Cryptoniscidae), female lateral view; Q) *Hemioniscus balani* Buchholz, 1866 (Hemioniscidae), juvenile female dorsal view; R) *Hemioniscus pagurophilus* Williams & Boyko, 2006 (Hemioniscidae), male dorsal view. (A, B, F, from [83]; C from [84]; D, E from [85]; G, H from [37]; I, J from [86]; K, L from [87]; M, N from [88]; O from [21]; P, Q from [8]; R from [28]). Scale bars: A, C, F = 1 mm; B, O, M = 0.5 mm; D, H, R = 0.25 mm; E = 0.075 mm; G = 2 mm; I, J = 0.3 mm; N = 0.15 mm; rest not to scale.

Members of Bopyridae are nearly all ectoparasitic on decapod hosts (Figure 1A–E), including infestation of some symbiotic hosts (i.e., representing hyperparasitism; Figure 1G). The bopyrid subfamilies Argeiinae, Bopyrinae, loninae, Orbioninae and Pseudioninae are found in the left or right branchial chambers of decapod hosts (or under the abdomens of hosts for a few species of Ioninae). Females of the bopyrid subfamily Pseudioninae (which mostly parasitize anomuran, brachyuran and caridean hosts) have a slightly modified form (Figure 3A), whereas the males are more like a generalized isopod (Figure 3B). Females are more modified in some of the branchial groups; for example, members of Ioninae (mostly parasites of brachyuran and mud shrimp hosts) often possess long lateral plates and pleopods with highly digitate margins (Figure 3C). The bopyrid subfamilies Athelginae, Hemiarthrinae (most species), and Phyllodurinae are ectoparasitic on the abdomens of hosts (hermit and king crabs, carideans and mud shrimp, respectively). Females of Athelginae are often symmetrical but with anterior pereopods clustered around the head and oostegites extending beyond the margin of the head (Figure 3D); males may have fused segmentation of the pleon (Figure 3E). In contrast, many females of Hemiarthrinae are highly asymmetrical with the brood pouch skewed to one side of the body (Figure 3F). The bopyrid subfamily Entophilinae is unique in being endoparasitic (one species in the visceral cavity of an anomuran host and one in the abdomen of a mud shrimp host). Females of these species have a head concealed by coxal plates that extend down the body and their pereopods are reduced with some articles fused (Figure 3G), whereas males are broad and subelliptical in shape and also possess reduced pereopods (Figure 3H).

Dajids are ectoparasitic on the cephalothorax, head, gills, or sometimes in the marsupium of euphausiid, mysid and shrimp hosts. Females are typically ovate in shape and highly modified, sometimes with claw-like pereopods or antennae [25] to clutch onto the eyestalks of hosts (Figure 3I), and the males may also be modified with fusion of the head and the first segment plus the pleonal segments (Figure 3J). Entoniscidae species are endoparasitic within the visceral cavity of anomuran, brachyuran and shrimp hosts, inducing the host to form a sheath within which the parasite resides [26] (Figure 1F). Entoniscid females are some of the most highly modified of the parasitic isopods, with reduced or absent pereopods but retaining a brood chamber (Figure 3K), whereas male entoniscids have distinct segmentation but also have reduced pereopods (Figure 3L).

Cryptoniscoidea contains seven families composed of endoparasitic species associated with a diverse assemblage of crustacean hosts and include some species that are hyperparasitic [21], [27], [28]. A single record of cryptoniscoid larvae on a non-crustacean host exits: an apparent case of accidental infection of a squid [29]. Female cryptoniscoids are the most highly modified parasitic isopods and usually are sac-like forms (Figure 1H), lacking pereopods, oostegites and, in some families, all segmentation, whereas the neotenous male cryptoniscoids retain the larval morphology [4], [26], [30]. Asconiscidae is represented by one species that is a parasite of mysids; Cabiropidae species parasitize isopods; Crinoniscidae species parasitize sessile and pedunculate thoracican barnacles; Cryptoniscidae species parasitize rhizocephalan barnacles on decapods and also some decapod hosts directly; Cyproniscidae species parasitize ostracods; Hemioniscidae species parasitize thoracican and acrothoracican barnacles; and species of Podasconidae parasitize amphipods. Females of Cabiropidae (Figure 3M) and Crinoniscidae (Figure 3O) lose all segmentation, and some species within the former group are hyperparasitic in the brood chamber of bopyrids. Females of Cryptoniscidae also lose all segmentation and have an anterior portion embedded in the host and external posterior portion (Figure 3P), an example of convergence with rhizocephalan barnacles [4]. In contrast, females of Hemioniscidae retain their anterior segmentation (Figure 3Q) via incomplete biphasic molting [31]. Male cryptoniscoids (Figure 3N, R) are morphologically indistinguishable from the cryptoniscus larval form [21].

### Reproduction and life histories

Unlike parasitic isopods in Cymothooidea that are monoxenous and produce manca larvae that parasitize fish hosts, epicarideans are heteroxenous, infesting two hosts during their life cycle. For all epicaridean species that have been investigated, a pelagic calanoid copepod acts as the intermediate host and another crustacean acts as the definitive host. Intermediate host specificity is poorly known, with little research having investigated any of the interactions between bopyrids and copepod hosts (e.g., [32], [33], [34]).

**Figure 4:**
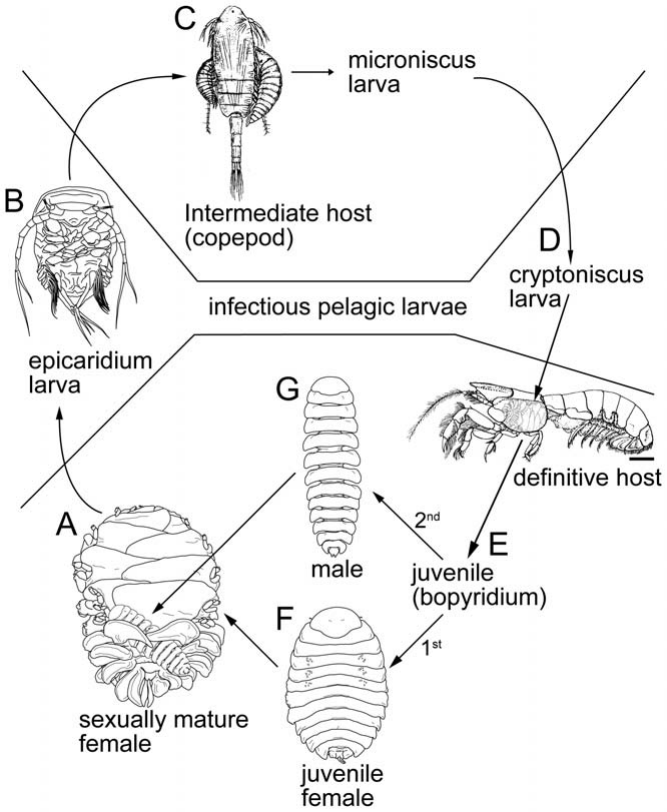
Epicaridean life cycle for the bopyrid isopod *Orthione griffenis* Markham, 2004. A sexually mature female and male in the gill chamber of the gebiid mud shrimp definitive host (*Upogebia pugettensis*). The female releases epicaridium larvae that parasitize calanoid copepod intermediate hosts. The epicaridium larva metamorphoses into a microniscus larva and then a cryptoniscus larva that settles onto a definitive mud shrimp host. The first juvenile isopod (bopyridium) to parasitize a host becomes female; subsequent isopods become male(s) and live on the female. Scale bar: 1 cm for definitive host (rest not to scale). From [72].

Sex determination is not fully known in epicarideans but can be genetically or epigametically controlled [35], [36]. Epigametic sex determination appears to be the case in many bopyroid species where the first isopod to settle becomes female and subsequent individuals become male(s) (Figure 4). The life cycle begins when the bopyrids reach sexual maturity on the definitive host and the male isopod fertilizes the eggs within the marsupium of the female (Figure 4A). The eggs give rise to epicaridium larvae (Figure 4B) within the marsupium, which are released in the water column where they parasitize copepod intermediate hosts (Figure 4C). The epicaridium larva pierces the body of the host, feeds on its blood, and metamorphoses into a microniscus larva. The microniscus larva feeds, eventually detaching from the copepod host and metamorphosing into a cryptoniscus larva (Figure 4D), the infective stage for the definitive crustacean host. The cryptoniscus larva settles and transforms into juvenile (bopyridium) (Figure 4E) that moves to the final attachment site on the host and, if female, will pierce the cuticle of the host and feed on its hemolymph or ovarian fluids [26]. Males reside on females and are not known to feed on hosts, although little is known of their feeding biology.

Species of the endoparasitic bopyroid subfamily Entophilinae create an exit pore near the base of the fourth pereopods of hosts and it is probably through this pore that larvae are released into the water [37] and then follow the typical bopyroid life cycle. Similarly, Entoniscidae females produce a posterior stalk that extends to the external environment of the host through the branchial region or eyestalks [38]. The life cycle of entoniscids is poorly known but it is suspected to also involve a copepod intermediate host and they presumably settle as cryptoniscid larvae in the branchial chamber and then enter hosts [39].

The life cycles of all Dajidae species are assumed to include pelagic copepod intermediate hosts, but this has only been confirmed in a small number of species [40]. For those that have been documented, the life cycle is similar to bopyrids and after development on the copepod host they settle onto definitive hosts as cryptoniscid larvae prior to maturation into males and females.

Cryptoniscoidea are protandric sequential hermaphrodites and, for those few species that have been studied, exhibit a similar life cycle to bopyrids [41]. However, as previously noted, the males of cryptoniscoids retain the cryptoniscus larval form [21].

### Feeding biology and impacts on hosts

Through their actions as hemolymph and ovarian fluid feeders, epicarideans can be parasitic castrators of their hosts. Some species of bopyrids and dajids are best considered partial parasitic castrators, since they have not been shown to wholly shut down reproduction of hosts [25], [38] and some bopyrids appear to have no effect on reproduction of female hosts. However, some bopyrids and dajids, as well as all Entoniscidae and Cryptoniscoidea, appear to be complete parasitic castrators. For example, females of Hemioniscidae attach to the ovaries of barnacle hosts and cause cessation of egg development although sperm development is not impacted [42]. Unlike rhizocephalans that castrate crustacean hosts via chemical means [4], epicarideans do so by the energy burden they impart on hosts [43], which can sometimes be compounded by multiple isopod infestations on a single host. In addition to these major potential impacts on host reproduction, parasitic isopods may also affect the morphology and perhaps also the behavior of hosts. For example, members of Bopyridae that branchially infest decapods cause large swellings of their branchiostegites (see [44]; Figure 1B). Cryptoniscoids can also cause swellings in certain hosts such as pedunculate barnacles (see [21], Figure 12A). Morphological impacts extend to changes in secondary sexual characteristics, including feminization of male hosts.

### Biodiversity and biogeography

Epicarideans are a diverse group, representing 7.7% (795/10,300) of described isopods. The rate description of parasitic species has not matched that of free-living isopods; in the late 1980s, approximately 13% of described isopods were epicarideans [5], [45]. This disparity will likely continue because, even though many undescribed epicarideans exist, many more free-living isopods are in need of description. For example, an estimated 600+ free-living species are undescribed from the Antarctic alone [46].

**Figure 5:**
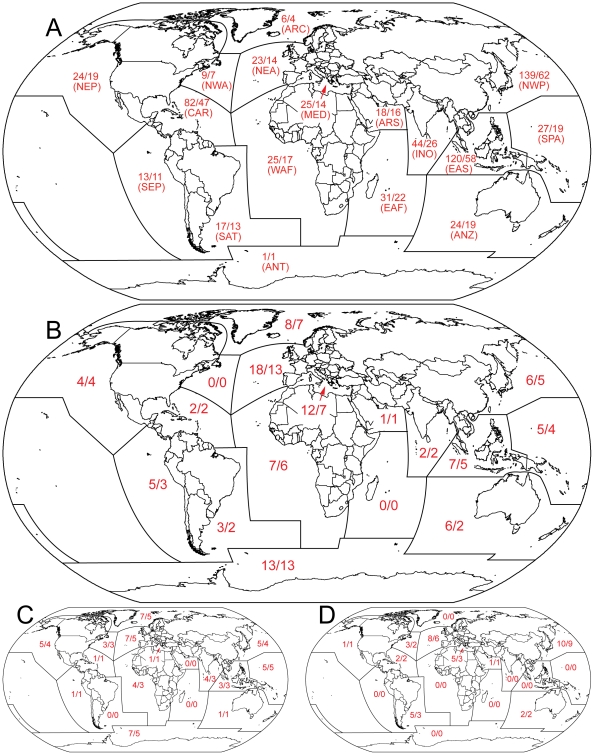
Zoogeographic distribution of parasitic isopods associated with crustacean hosts; numbers of species/genera shown within marine ecoregions. A) Distribution of Bopyridae. B) Distribution of Cryptoniscoidea. C) Distribution of Dajidae. D) Distribution of Entoniscidae. Ecoregional abbreviations, shown in parentheses in part A (ANT, Antarctic; ANZ, Australia/New Zealand; ARC, Arctic; ARS, Arabian Sea; CAR, Wider Caribbean; EAF, East Africa; EAS, East Asian Sea; INO, Central Indian Ocean; MED, Mediterranean; NEA, North East Atlantic; NEP, North East Pacific; NWA, North West Atlantic; NWP, North West Pacific; SAT, South Atlantic; SEP, South East Pacific; SPA, South Pacific; WAF, West Africa). Only described parasite species included. Ecoregions based on [15].

Of these 795 described parasitic isopods [14], the bulk of the diversity (76.1%; 605/795) belongs to Bopyridae (Figure 5A), followed by Cryptoniscoidea (12.5%; 99/795) (Figure 3B), Dajidae (6.8%; 54/795) (Figure 5C), and Entoniscidae (4.7%; 37/795) (Figure 5D). Within the Bopyridae, the numbers of species in each subfamily are currently: Argeiinae (12 species), Athelginae (41 species), Bopyrinae (118 species), Entophilinae (2 species), Hemiarthrinae (55 species), Ioninae (105 species), Orbioninae (38 species), Phyllodurinae (1 species) and Pseudioninae (232 species), with 1 species described from only the larval stage being considered *incertae sedis*. Bopyrids exhibit highest diversity within the North West Pacific (139 species), East Asian Sea (120 species), and Central Indian Ocean (44 species). However, we predict that the Central Indian Ocean and the East Asian Sea (in particular, the Indo-Malay-Philippines Archipelago) hold a wealth of undescribed species, reflecting our knowledge of host diversity patterns [47]. Predictions of more species in areas of the Indo-West Pacific are supported by recent discoveries in China ([48]), Australia [49] and the Philippines [50]. For example, in China approximately 13% (18 of 139) of bopyrid species (Figure 5A) have been described after 2005, and many additional new host and country records have been reported [51]. Our own collections from the Philippines have led to the discovery of at least 5 new species of bopyrids on shallow water hermit crabs (Williams & Madad, unpublished, Williams & Boyko, unpublished). The bopyrid fauna of the Indo-West Pacific may be more than two times greater than is presently known from that region [5].

The diversity patterns of Cryptoniscoidea are distinct from bopyrids, with the greatest diversity of cryptoniscoids at the high and low latitudes. Specifically, cryptoniscoids exhibit highest diversity in the North East Atlantic (18 species) and, when combined with the Arctic (8 species), >26% are found in this region of the northern hemisphere; the other areas of high diversity are the Antarctic (13 species) and Mediterranean (12 species) (Figure 5B). Similarly, Dajidae exhibits high diversity in the Antarctic (7 species) with 14 species in the Arctic and North East Atlantic regions combined (Figure 5C). Entoniscidae shows highest diversity in the North West Pacific (10 species) and North East Atlantic (8 species) but, surprisingly, there are no species described from the biodiverse East Asian Sea or Central Indian Ocean (Figure 5D). This “absence” is likely a reflection of the fact that entoniscids are all endoparasitic and their hosts typically need dissection for parasites to be detected [18]. East Africa (including Madagascar) has no recorded species of cryptoniscoids, dajids, or entoniscids, but this region is likely to contain examples of all three groups as many suitable hosts occur there. Lack of species in a region as a result of lack of sampling, rather than reflecting real biogeographic patterns, has been demonstrated for bopyrids [18], [52].

Most epicarideans are known from relatively shallow waters, although two described species of bopyrids are known from depths below 4000m, with undescribed species known down to 5210 m [18]. Distribution patterns of epicarideans must be influenced by a combination of both definitive (either benthic or pelagic) and intermediate (pelagic copepod) host distributions but few data are available to address the underlying mechanisms of distribution.

### Phylogeny and historical patterns

The epicarideans are currently divided into Cryptoniscoidea and Bopyroidea within Cymothoida [53]. Whereas some cryptoniscoids parasitize decapod hosts, most (ca. 90%) are known from other crustaceans, such as peracarids, ostracods, and cirripedes, although a number of taxa, principally from the Antarctic, have no known hosts because they were described solely from planktonic larval stages. Species of Bopyroidea, in contrast, are known almost entirely from decapod hosts. With 605 described species, Bopyridae is the most speciose family in Bopyroidea, as well as the second most speciose family of isopods after Sphaeromatidae (ca. 630 species). The two other families of Bopyroidea are Entoniscidae (37 species), which are endoparasites of decapods, and Dajidae (54 species), which are ectoparasites of shrimp, mysids, and euphausids. As with the Bopyroidea+Cryptoniscoidea grouping (="Epicaridea” of authors), Bopyridae+Entoniscidae+Dajidae has long been assumed to be monophyletic, based in large part on reproductive biology, life cycles, and the morphology of the males; but no cladistic phylogenetic analyses have ever been conducted for these taxa.

Currently, Bopyridae is divided into nine subfamilies [4]. The characters used most often to define species and higher taxa come primarily from female morphology in Bopyroidea species, although males display characters useful in generic conceptualization. This is in contrast to the situation in Cryptoniscoidea, where the most useful characters are those of the paedomorphic males, because the females are so highly reduced in overall morphology.

In the subfamilies Pseudioninae, Bopyrinae, Argeiinae and Orbioninae, the adult female parasite is located on the decapod host in the right or left branchial chamber. The branchial chamber is also the usual site of attachment for members of Ioninae, but species of *Rhopalione* are found under the abdomens of their pinnotherid hosts. In Athelginae, the females are located on the dorsal abdomen of the host hermit or king crab, while in Phyllodurinae, the female isopod is situated on the ventral surface of the *Upogebia* (Gebiidea) host abdomen. Female isopods of Hemiarthrinae are found either on the dorsal or ventral surface of the abdomen, laterally on the carapace, or, uniquely for one species, inserted into the buccal region of the host shrimp [8]. The two species of Entophilinae are similar in habitat to entoniscid isopods, living as endoparasites in the thoracic or abdominal regions of their galatheid and gebiidean hosts.

Until recently, no phylogenetic testing of the monophyly of any epicaridean taxa has ever been attempted using morphological or molecular data, although non-cladistic hypotheses of subfamilial relationships exit [5], [54], [55]. Pseudioninae was considered basal in all studies, but under one conception the abdominal parasitic subfamilies Athelginae and Hemiarthrinae were considered sister taxa, and two lineages were derived from Pseudioninae [54], [55], whereas another conception proposed four lineages arising from Pseudioninae and placed Athelginae and Hemiarthrinae on two different branches [5]. Examination of co-evolution between bopyrids and their definitive hosts, based on these conceptions of bopyrid evolution, suggests a high degree of incongruence, with frequent host switching (i.e., horizontal transfer) occurring [4]. However, the purported basal bopyrid subfamily (Pseudioninae) is only questionably monophyletic and may obscure these results. Clearly, more analyses are needed at all levels for these taxa. It is important to note that the choice of exemplar taxa must be made carefully, as many of the more speciose genera of bopyrids may be paraphyletic, based on observations of morphology. Our own ongoing work using 18s rRNA data across Bopyroidea and Cryponiscoidea indicates a pattern of evolution considerably different from the earlier studies, with both Bopyridae and Bopyroidea presenting as non-monophyletic taxa, with multiple occurrences of host switching over time (Boyko et al., in prep.).

### Human related issues

Parasitic isopods impact a variety of commercially important hosts, including brachyuran crabs, false kings crabs, king crabs, and shrimp (e.g., [56], [57], [58], [59], [60]) or are prey for commercially important species (e.g., [61]). Although bopyrids do not pose a medical threat to humans, their presence in the branchial chamber of hosts can negatively impact salability of infected hosts such as shrimp [62]. The parasites can shut down reproduction of hosts but most host populations do not appear to be strongly impacted, as the parasites are typically found in low prevalence [60], [63]. However, some host populations have been shown to have high prevalence of bopyrid isopods, such as *Argeia pugettensis* (Argeiinae) that infests at least 20 species of crangonid shrimp hosts, some of which are commercially important [64], [65]. Parasitic isopods can also be found on shrimp sold in the aquarium trade [66] and used as bait [67].

Although many native parasites do not markedly impact host populations, introduced parasitic isopods have been shown to cause severe negative impacts on host populations. For example, the bopyrid *Orthione griffenis* Markham, 2004 (Pseudioninae) infests the gebiid mud shrimp *Upogebia pugettensis* along the west coast of the United States. This parasite was apparently introduced from Asia sometime during the late 1980s [68], [69], [70], [71], [72], [73]. The parasite is thought to have caused the observed collapse of host populations on mudflats along the North West Pacific subsequent to its introduction [68], [73]. This parasite has significant ecological and economic implications for humans because the host mud shrimp is an ecosystem engineer and has impacts on bivalve fisheries through its activities in influencing sedimentation [74], [75], [76].

Parasitic isopods have been evaluated for use in a variety of applied contexts. For example, some parasitic isopods have been examined as potential biological controls for introduced host decapods [77], [78], [79] [77], [78], [79] [76], [77], [78] [72], [73], [74] and hyperparasitic isopods considered as biological controls for bopyrids on penaeid shrimp [80]. However, because of their indirect life cycle and potential to invade non-target hosts, the use of epicaridean parasites as biological controls requires careful study. Parasitic isopods also have been used as biological indicators of disturbed habitats [81] and may make hosts more susceptible to environmental toxins [82].
